# The efficacy and safety of a combined multipolar radiofrequency with pulsed electromagnetic field technology for the treatment of vaginal laxity: a double-blinded, randomized, sham-controlled trial

**DOI:** 10.1007/s10103-021-03438-3

**Published:** 2021-10-14

**Authors:** Penpun Wattanakrai, Nattawan Limpjaroenviriyakul, Darin Thongtan, Rujira Wattanayingcharoenchai, Jittima Manonai

**Affiliations:** 1 Ramathibodi Laser Center, Division of Dermatology, Department of Internal Medicine, Faculty of Medicine, Ramathibodi Hospital, Mahidol University, Bangkok, 10400 Thailand; 2 Division of Female Pelvic Medicine and Reconstructive Surgery, Department of Obstetrics and Gynecology, Ramathibodi Hospital, Mahidol University, Bangkok, 10400 Thailand

**Keywords:** Vaginal laxity, Vaginal rejuvenation, Radiofrequency, Pulsed electromagnetic field, Sexual function

## Abstract

Non-invasive vaginal rejuvenation with radiofrequency (RF) and lasers devices have gained popularity, but well-designed studies confirming their effectiveness are lacking. The aim of this study was to compare the efficacy and safety of a multipolar RF and pulsed electromagnetic field-based device (PEMF) versus sham for vaginal laxity. Thirty-two premenopausal females with ≥ 1 vaginal delivery and self-reported vaginal laxity were randomized into 2 groups: active (RF + PEMF) and sham. Both groups received 3 vaginal treatments at 3-week interval. The Vaginal Laxity Questionnaire (VLQ), perineometer measurements, and Brink score were conducted at baseline, 4, and 12 weeks after treatments. Pre and post-treatment vaginal histology, Female Sexual Function Index (FSFI), subjects’ satisfaction, pain, and adverse events were assessed. The active group VLQ scores increased and were significantly better than the sham group (*p* < 0.001). At the final follow-up, 50% of the active group reported no vaginal laxity (VLQ > 4) versus 12% in the sham group (*p* = 0.054). In the active group, all domains of perineometer measurements and Brink scores (*p* < 0.001), FSFI scores (*p* < 0.05), and patients’ satisfaction (*p* < 0.001) were significantly increased and higher in the active group. Mild adverse effects including pain and burning sensation were not different between groups except for itch which was significantly higher in the sham arm (*p* = 0.014). Histology after RF + PEMF treatments demonstrated neocollagenesis, neoelastogenesis, and neoangiogenesis. In conclusion, combination RF + PEMF therapy was safe, improved vaginal laxity, strengthened pelvic floor muscles, and improved female sexual function for at least 12-week post-procedures with confirmed histological improvements. This study was registered on the Thai Clinical Trials Registry, TCTR20200803002 on 2020–07-30 “retrospectively registered.”

## Introduction

Vaginal introital laxity is a common consequences of pregnancy, vaginal childbirth, and connective tissue changing due to aging [1, 2]. However, this condition is frequently underreported due to embarrassment and lack of recognition [3, 4]. Vaginal laxity (VL) may result in loss of physical and sexual sensation during intercourse leading to a negative impact on female sexual function, self-image, and quality of life [5–8].

Many treatment modalities for VL exist ranging from Kegel exercises and electrical stimulation to promote perineal muscle strength, pharmacological therapy to surgical and non-surgical procedures [9–13]. Surgical options can be performed to improve vaginal introitus tightness but are associated with pain, significant risks, and post-operative downtime [11, 12]. The use of non-invasive, energy-based devices including radiofrequency (RF) and lasers as a non-surgical option for treatment of vaginal laxity (VL), vulvovaginal atrophy (VVA) or genitourinary syndrome of menopause (GSM), orgasmic dysfunction, and stress urinary incontinence (SUI) has recently gained popularity. Both laser and RF treatments induce neocollagenesis, neoelastogenesis, and neovascularization in the submucosa which may contribute to improved tightness of the vaginal canal and may also improve sensitivity of vulvovaginal tissues. The mechanism is believed to be secondary to activation of heat shock proteins and triggering of the inflammatory and proliferative cascade [13–16].

Unlike lasers, which involve selective photothermolysis of specific chromophores causing ablation and thermal coagulation of tissue, RF produces an electromagnetic wave that generates heat through tissue impedance in the vaginal tissue without disruption of the epidermal-dermal barrier. Based on their number of electrodes, non-invasive RF devices can be categorized as monopolar, bipolar, tripolar, quadripolar, and multipolar (with or without cooling systems) [13–16]. In the same device, RF can be combined with pulsed electromagnetic fields (PEMF) which has been used for non-ablative skin tightening, facial rejuvenation, and treatment of cellulite and striae [17–20]. PEMFs are induced by short pulses of electrical current that penetrates into the tissue and results in non-thermal stimulation of molecular and cellular activities. PEMF increases collagen production by dermal fibroblasts, stimulates growth factor synthesis, and angiogenesis, leading to its wound-healing effects [18, 21, 22].

The primary objective of this study was to evaluate the efficacy and safety of a combined multipolar RF with PEMF device for the treatment of VL. The secondary objective was to determine the treatment satisfaction, pelvic floor muscle strength, and sexual function in both groups.

## Materials and methods

### Study design

This is a prospective, randomized, sham-controlled, double-blinded study approved by the ethics committee of Ramathibodi Hospital, Mahidol University certificate MURA2018/815 and registered on the Thai Clinical Trials Registry, TCTR20200803002. The study protocol conformed to the ethical guidelines of the 1975 Declaration of Helsinki and all its revisions. Written informed consent was obtained from subjects before enrollment.

### Study population

Thirty-two healthy premenopausal females were enrolled according to the exclusion and inclusion criteria. Inclusion criteria consisted of females aged 20–45 years old with at least 1 vaginal delivery and self-assessment of vaginal laxity by a Vaginal Laxity Questionnaire (VLQ) score no higher than 3 on the (defined as “very loose”, “moderately loose”, or “slightly loose”) [12, 23, 24]. Other inclusion criteria include normal genito-pelvic examination and Papanicolaou smear cytology, negative pregnancy test, willingness to participate in vaginal intercourse at least two times per month, and be either surgically sterilized or willing to use an acceptable method of birth control at least 1 month before screening and throughout the study duration. Exclusion criteria included patients with implanted medical devices, copper intrauterine device, Pelvic Organ Prolapse Quantification System (POP-Q) stage ≥ 2, history of chronic vulvar pain, unexplained vaginal bleeding, concurrent sexual transmitted disease/pelvic inflammatory disease or infection, previous surgery or intervention for treatment of VL, and other concomitant illnesses or medications affecting wound healing or sexuality.

### Randomization and treatment procedure

Subjects were randomized 1:1 into 2 groups: active (RF + PEMF) and sham group using a computer-generated block randomization scheme with a block size of 4. Both groups received three vaginal treatments scheduled at 3-week intervals at least 7 days after cessation of their menstrual cycle. Procedures were performed by trained physical therapists not involved in any other parts of the study assessments. Subjects were treated with a multipolar RF and PEMF-based device (Venus Fiore™, Venus Concept, San Jose, CA). This device is equipped with an internal handheld applicator and two external applicators (for treatment of skin laxity of the mons pubis and the labia). Only the internal applicator was evaluated. The internal cylindrical applicator contains 3 integrated temperature sensors and 3 pairs of bipolar electrodes that deliver RF and PEMF circumferentially throughout the entire vaginal canal. There is a 15-mm circumferential gap housing no electrodes that is positioned directly below the urethra during a procedure. The device has an automatic temperature control (ATC) feature that offers both the capability to maintain predetermined temperature, as well as to adjust temperature and energy delivery on each pair of electrodes independently. While the device is operated in the ATC mode, the system reads the local temperature twenty-five times per second (every 40 ms) and adjusts the RF output five times per second (every 200 ms) to maintain the desired temperature. Water soluble ultrasound transmission gel (Aquasonic, Parker Laboratories, Fairfield, NJ) was applied as RF coupling media and lubricant. During treatment, the internal applicator was fully inserted into the vaginal canal and held stationary throughout the treatment period of approximately 15 min. Local anesthesia is not required. After the procedure, patients were requested to avoid sexual intercourse for at least 24 h.

**Fig. 1:**
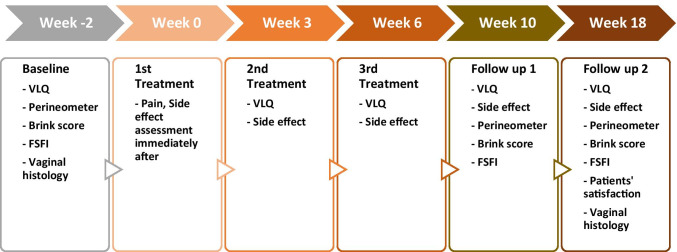
Timeline of the study protocol

The active group received treatment settings at an RF energy level of 50–60% (of 80 watts, 1 MHz maximum) target therapeutic temperature level of 41–44 °C with the PEMF turned on (pulsed magnetic field flux 15 gauss) for the proximal pair of electrodes (closest to the applicator base or labia). For the mid and distal pairs of electrodes (furthest from the applicator base or closest to the cervix), the energy level was adjusted to 65% target therapeutic temperature level of 42–45 °C. The Sham group received a nontherapeutic RF energy level of 1% with PEMF turned off, for all three pairs of electrodes. To allow the subjects and investigators to remain blinded, all subjects were treated and followed exactly the same throughout the study, regardless of their assigned randomization group. Follow-up visits were scheduled at 4 weeks and 12 weeks after completing the 3rd treatment. The study flowchart is shown in Fig. 1.

### Histological analysis

Six subjects (3 from each group) had consented to undergo pre- and post-treatment biopsies for histological evaluation. Three-mm punch biopsies from the vaginal wall were obtained 2 weeks before the initial treatment and 12 weeks after completing three sessions. Formalin fixed vaginal tissues were stained with hematoxylin and eosin (H&E) and stained for elastic fibers using Verhoeff-van Gieson stain. Specimens were evaluated by a single dermato pathologist blinded to the intervention and sequence of the biopsy specimen.

#### Subjective assessments

To assess the primary study outcome objective, subjects completed the Vaginal Laxity Questionnaire (VLQ) at baseline week 0, 3, and 6 and at 4-week and 12-week follow-up visits. The VLQ is a 7-point Likert scale that has been used for self-reporting of the level of VL in clinical studies and is defined as follows: very loose (score = 1), moderately loose (score = 2), slightly loose (score = 3), neither loose nor tight (score = 4), slightly tight (score = 5), moderately tight (score = 6) or very tight (score = 7). “No vaginal laxity” was classified as a VLQ score greater than 4 (> 4).

Secondary efficacy assessments of sexual function was evaluated using a certified Thai translation of the Female Sexual Function Index (FSFI) [25] at baseline and at 4 and 12 weeks after the 3rd treatment. The FSFI is a validated 19-item questionnaire divided into six domains of female sexual function: desire, arousal, lubrication, orgasm, satisfaction, and pain. The domain scores are combined to create a total score range of 2 to 36. A total FSFI score ≤ 26.55 is recognized in the medical literature as indicating sexual dysfunction [26]. The VLQ and FSFI have been used in clinical studies of RF treatment for VL [12, 23, 24].

Pain score during the procedure was rated immediately after the 1st session using a 10-point visual analog scale (VAS) (0 = no pain to 10 = severe pain). At the final visit, subjects were asked to grade their overall satisfaction of the intervention with the VAS 0 = not satisfied to 10 = most satisfied. Adverse reactions after each session were recorded and graded as mild, moderate, and severe.

#### Objective assessments

Perineometer or vaginal manometer is an instrument used to measure the strength of voluntary contractions of the pelvic floor muscles. Perineometer measurements of maximum pressure (mmHg), average pressure (mmHg), and total duration (seconds) were recorded for 3 consecutive pelvic floor muscle contractions. The Brink scale [27] is a commonly used digital assessment of pelvic floor muscle strength performed by inserting 2 fingers into the vagina and asking the subject to perform a single Kegel contraction. The contraction is scored using three separate 4-point rating scales for pressure, vertical finger displacement, and duration which are then summed to a score ranging from 3 to 12: 3–6 = very weak, 7–9 = strong, and 10–12 = very strong. Brink and perineometer assessments were done at baseline, 4, and 12 weeks follow-up by the same specialized uro-gynecology nurse blinded to the treatments.

### Statistical analyses

Sample size and statistical data analysis are calculated by using Stata version 14 (StataCorp. 2015. Stata Statistical Software: Release 14. College Station, TX). The sample size was estimated from a previous study based on the increase of the mean VLQ scores compared to baseline by 1 between the two interventions (active RF + PEMF and sham), with type I error α of 0.05 and type II error β of 0.20 [28]. The sample size was determined to be 15 participants per group. Sixteen subjects were enrolled in each group to cover 5% dropout.

Categorical variables and continuous variables were calculated as frequency (percentage) and mean, standard deviation (SD), and standard error of the mean (SE) for descriptive statistical analyses. For demographic data, independent *t*-test was used for age, weight, BMI, VLQ score, perineometer, Brink score, and FSFI, whereas Fisher’s exact was used for number of vaginal deliveries. The outcome data was adjusted to the baseline by using a linear combination of the parameters before inferential statistical analysis comparing the outcomes between groups, which include VLQ score, perineometer, Brink score, and FSFI. Student’s paired *t*-test and Wilcoxon signed-ranks test for matched pairs were used to test for changes between baseline and follow-up visits. Patient satisfaction was analyzed using independent *t*-test. Adverse events were analyzed by Fisher’s exact and Pearson’s Chi-square where applicable. *p* Values < 0.05 were considered statistically significant. Unless otherwise stated, the mean and SE were reported.

## Results

All of the 32 enrolled female subjects completed the study protocol and were included in the safety and efficacy analysis. Baseline characteristics are shown in Table 1.

**Table 1:** Baseline demographic data

Demographic data	Active group (*n* = 16)	Sham group (*n* = 16)
Age (years ± SD) (range)	37.19 ± 5.33 (30–45)	35.5 ± 4.97 (26–43)
Weight (kg ± SD)	57.53 ± 6.36	60.22 ± 10.48
BMI (kg/m^2^ ± SD)	23.43 ± 2.93	24.07 ± 3.97
Vaginal deliveries (*N* (%))		
0	0 (0%)	0 (0%)
1	5 (31.25%)	6 (37.5%)
2	10 (62.5%)	9 (56.25%)
3	1 (6.25%)	0 (0%)
4	0 (0%)	1 (6.25%)
Baseline VLQ score (1–7; mean ± SD)	2 ± 0.63	2.12 ± 0.34
Baseline perineometer		
Maximum pressure (mmHg ± SD)	20.19 ± 14.14	24.29 ± 12.84
Mean pressure (mmHg ± SD)	17.11 ± 11.33	20.17 ± 10.76
Duration (seconds ± SD)	4.86 ± 1.15	3.63 ± 2.39
Baseline Brink score (3–12; mean ± SD)	8.56 ± 2.97	8.75 ± 2.54
Baseline FSFI (2–36; mean ± SD)	22.78 ± 4.5	24.0 ± 3.1

### VLQ score

**Fig. 2:**
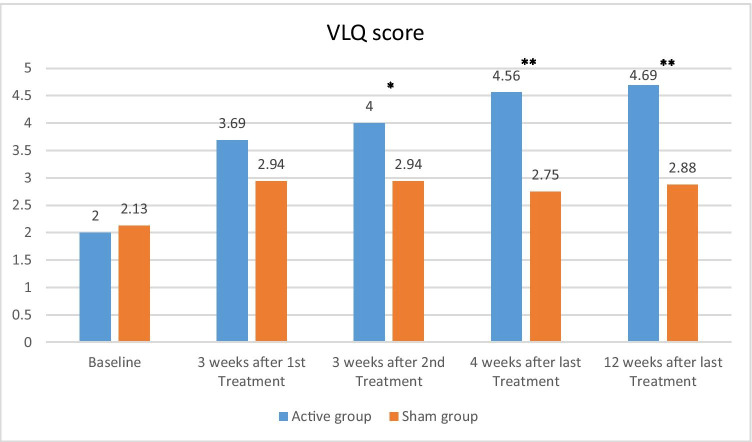
Mean Vaginal Laxity Questionnaire (VLQ) scores at baseline, 3 weeks after 1 treatment, 3 weeks after 2 treatments, and 4 weeks and 12 weeks after 3 treatments. *p value < 0.05, **p value < 0.001

The baseline mean VLQ scores of both groups were not significantly different (*p* = 0.75, Table 1). The mean VLQ scores in the active group improved from baseline 2.00 ± 0.63 to 4.69 ± 1.35 at the 12-week follow-up visit, compared to sham group baseline 2.13 ± 0.34 to 2.88 ± 1.26, respectively (Fig. 2). Within group comparisons, both interventions significantly improved the mean VLQ scores when compared to baseline (*p* < 0.05), but the degree of improvement was greater in the active group which achieved 128% improvement compared to baseline (*p* < 0.001) and 134% (*p* < 0.001) at 4 weeks and 12 weeks follow-up versus the 29% and 35% improvements in the sham group, respectively.

When comparing between interventions, the active group showed statistically significant higher mean VLQ score compared to sham group as early as 3 weeks after the 2nd treatment with VLQ values 4.0 ± 1.41 and 2.94 ± 1.06, respectively (*p* = 0.007), and the results were maintained significantly throughout the 4- and 12-week follow-up visits 4.56 ± 1.26 versus 2.75 ± 1.13 (*p* < 0.001) and 4.69 ± 1.35 versus 2.88 ± 1.26, respectively (*p* < 0.001).

Fifty percent (8 of 16 subjects) in the active group reported no VL (VLQ score > 4) at 12 weeks after treatments compared to 12% (2 of 16 subjects) in the sham group (*p* = 0.054). All of the active group participants reported an improvement in VLQ score of at least one point, with 8 of 16 subjects (50%) achieving improvement of three or more levels, whereas 8 subjects (50%) in the sham group showed no VLQ score improvement.

### Perineometer measurements

When compared to baseline, the active intervention significantly improved in all three parameters of the perineometer measurements (*p* < 0.001) at both follow-ups, while the sham group showed no significant improvement from baseline at either visits (Table 2).

**Table 2:** Comparison of the outcomes between active group and sham group

Parameter		Active group		Sham group		*p* Value*
			*p* Value^+^		*p* Value^++^	
Perineometer	Maximum pressure (mmHg) Baseline 4-Week follow-up 12-Week follow-up	20.19 ± 3.54 27.24 ± 3.60 28.58 ± 3.44	- < 0.001 < 0.001	24.29 ± 3.21 24.41 ± 3.34 23.39 ± 3.08	- 0.822 0.166	0.909 < 0.001 < 0.001
	Mean pressure (mmHg) Baseline 4-Week follow-up 12-Week follow-up	17.11 ± 2.83 22.76 ± 3.10 23.99 ± 3.13	- < 0.001 < 0.001	20.17 ± 2.69 19.09 ± 2.48 19.15 ± 2.55	- 0.152 0.118	0.970 < 0.001 < 0.001
	Duration (seconds) Baseline 4-Week follow-up 12-Week follow-up	4.86 ± 0.29 5.90 ± 0.39 6.37 ± 0.41	- < 0.001 < 0.001	5.63 ± 0.60 5.95 ± 0.36 6.27 ± 0.42	- 0.570 0.243	0.512 0.226 0.139
Brink score	Baseline 4-Week follow-up 12-Week follow-up	8.56 ± 0.74 9.25 ± 0.64 9.69 ± 0.62	- 0.016 0.006	8.75 ± 0.64 8.88 ± 0.62 9.06 ± 0.61	- 0.164 0.056	0.938 0.050 0.465
FSFI	Baseline 4-Week follow-up 12-Week follow-up	22.78 ± 1.14 25.23 ± 0.66 26.26 ± 0.83	- 0.022 0.002	24.0 ± 0.76 23.73 ± 0.69 24.94 ± 0.85	- 0.452 0.033	0.586 ± 0.016 ± 0.020 ±
Patients’ satisfaction (0–10)		8.37 ± 0.25	-	5.79 ± 0.31	-	< 0.001

^*^p Value for the change between baseline and follow-up for the active group versus the sham group

^+^*p* Value between baseline and follow-up in active group

^++^*p* Value between baseline and follow-up in sham group

#### Maximum pressure

**Fig. 3:**
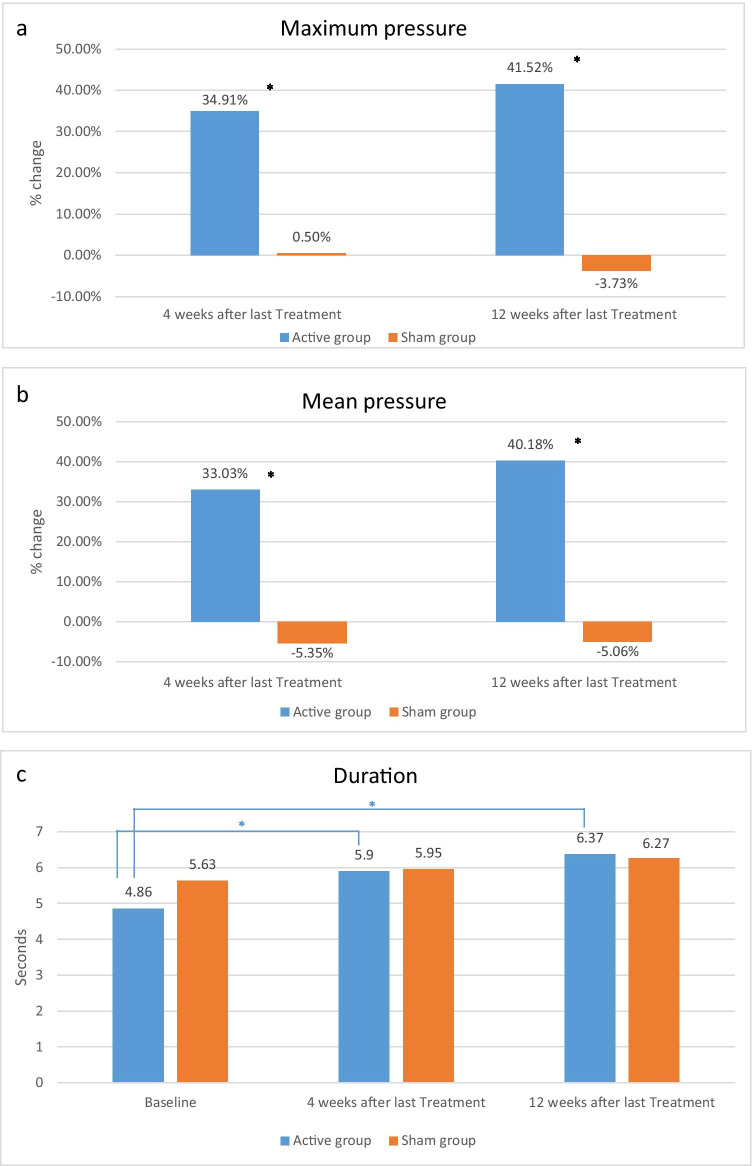
Perineometer measurements on each visit. **A** Percentage change of maximum pressure compared to baseline at 4-week and 12-week post-treatment; *p value < 0.005. **B** Percentage change of mean pressure compared to baseline at 4-week and 12-week post-treatment; *p value < 0.005. **C** Mean duration of voluntary vaginal contraction comparing baseline, 4-week, and 12-week post-treatment; *p value < 0.001

In comparison between the two interventions, the maximum pressure in active group was significantly better than the sham group at the 4-week follow-up 27.24 ± 3.6 versus 24.41 ± 3.34, respectively (*p* < 0.001) and at 12-week follow-up 28.58 ± 3.34 versus 23.39 ± 3.08, respectively (*p* < 0.001)**.** The percentage of improvements in the active group were significantly better, 34.92% and 41.56% at both follow-ups over baseline, compared to the sham group which were 0.50% and − 3.71%, respectively (*p* < 0.005) (Fig. 3A).

#### Mean pressure

The mean pressure significantly increased from baseline only in the active arm (*p* < 0.001). Comparing between interventions, the active group results were significantly better than the Sham group (Table 2). At 4-week follow-up, mean pressure in active group versus sham group was 22.76 ± 3.10 and 19.09 ± 2.48, respectively (*p* < 0.001) and 23.99 ± 3.13 and 19.15 ± 2.55, respectively, at 12 weeks (*p* < 0.001). The percentage changes of mean pressure over baseline in the active group were increased at 33.03% and 40.18% at 4- and 12-week follow-up, respectively, in contrast to the − 5.35% and − 5.06% decrease in the sham group (Fig. 3B).

#### Duration

The active group mean duration of pelvic contractions significantly increased from baseline (4.86 ± 0.29) at 4-week (5.90 ± 0.39; *p* < 0.001) and 12-week follow-up (6.37 ± 0.41; *p* < 0.001), while there was no significant improvement in the sham group durations. However, there were no significant differences in the duration between the two interventions at either measurements (Table 2, Fig. 3C).

### Brink scale

**Fig. 4:**
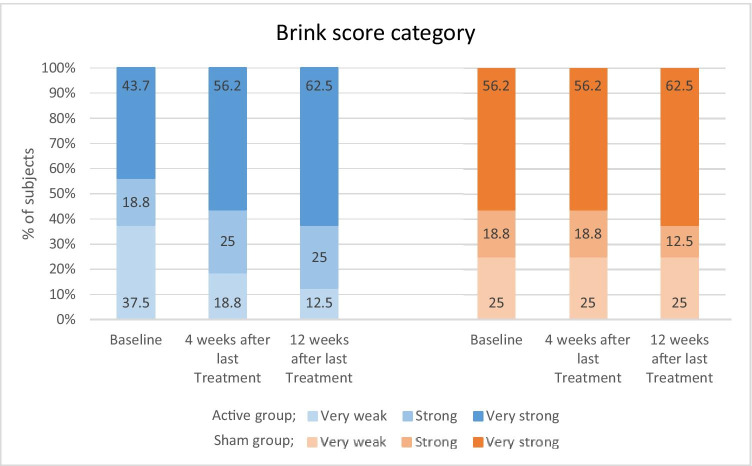
Comparison of percentage of subjects in each Brink score category; very week (3–6), strong (7–9), and very strong (10–12) baseline compared to 4-week and 12-week post-treatment

Mean Brink score in the active group was 9.25 ± 0.64 and 9.69 ± 0.62 at 4-week and 12-week follow-up, respectively, versus in 8.88 ± 0.62 and 9.06 ± 0.61, respectively, in the sham group. There was no significant difference between group at either visit (*p* = 0.05 and *p* = 0.46). However, in the active group, the Brink score statistically significantly increased from 8.56 ± 2.97 (baseline) to 9.25 ± 2.57 (*p* < 0.001) and 9.69 ± 2.47 (*p* < 0.001) at 4-week and 12-week follow-up, whereas the sham group saw no significant improvement within the group (Table 2). When comparing the proportion of the 3 Brink score categories, the very weak scores in the sham group did not change from 25% baseline through follow-ups versus the reduction from 37.5% at baseline to 18.75% and 12.5% in the active group follow-up, respectively. Conversely, the percentage of subjects with very strong Brink scores in the active group increased from 43.75% (baseline) to 56.25% and 62.5% at follow-ups representing an 18.75% increase versus 6.25% increase in the sham group (56.25% to 62.5%, respectively) (Fig. 4).

### FSFI score

**Fig. 5:**
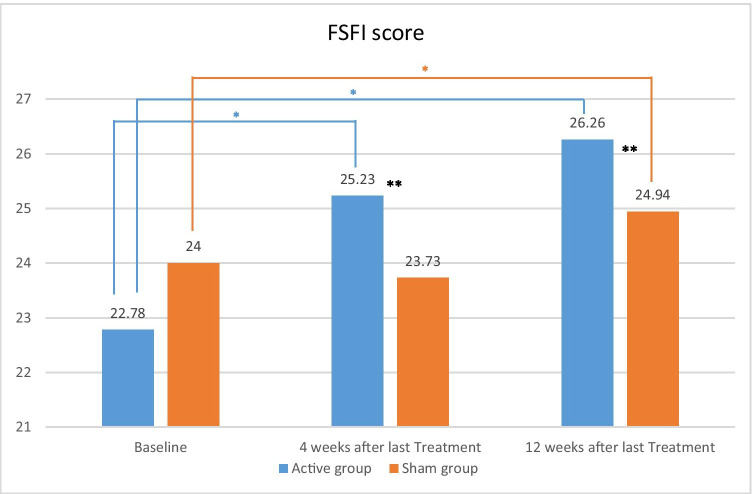
Mean scores for the combined FSFI domains (desire, arousal, lubrication, orgasm, satisfaction, and pain) at baseline, 4-week, and 12-week post-treatment. *Statistically significant difference over baseline; *p* value < 0.05. **Statistically significant difference between groups; *p* value < 0.05

In the active group, the mean FSFI total score significantly increased from baseline (22.78 ± 1.14) to 25.23 ± 0.66 (*p* < 0.05) and 26.26 ± 0.83 (*p* < 0.005) at both follow-up visits, while the mean FSFI total scores in the sham arm were not statistically different from baseline (24.00 ± 0.76) at the 4-week follow-up (23.73 ± 0.69) but significantly increased at the last follow-up (24.94 ± 0.85 (*p* < 0.05)) (Table 2, Fig. 5).

#### Subjects’ satisfaction

The mean VAS score for overall satisfaction of the treatments in the active group was significantly higher 8.37 ± 0.25 versus 5.79 ± 0.31 in the sham group (*p* < 0.001) (Table 2).

### Safety

The mean VAS scores for pain and burning sensation were not significantly different between interventions. The VAS for pain was 0.50 ± 0.30 and 0.38 ± 0.18 in active and sham group, respectively (*p* = 0.75), while the VAS for burning was 0.75 ± 0.37 and 0.31 ± 0.15 in the active and sham group, respectively (*p* = 0.22). Adverse effects assessed from the total of 48 treatments in each group are shown in Table 3. Pain, burning sensation, pain during sexual intercourse, and burning sensation during sexual intercourse were not significantly different except for itching which was significantly higher in the sham group (20.83%) compared to the active group (4.17%) (*p* = 0.014) as shown in Table 3. All adverse effects were mild.

**Table 3:** Adverse effects from all treatment sessions in both intervention groups

Adverse effects	Active group (*n* = 48 sessions) *n* (%)	Sham group (*n* = 48 sessions) *n* (%)	*p* Value
Pain			
-Yes	6 (12.5)	2 (4.17)	0.14
-No	42 (87.5)	46 (95.83)	
Burning sensation			
-Yes	1 (2.08)	2 (4.17)	0.557
-No	47 (97.92)	46 (95.83)	
Itching			
-Yes	2 (4.17)	10 (20.83)	0.014*
-No	46 (95.83)	38 (79.17)	
Pain during sexual intercourse			
-Yes	12 (25)	7 (14.58)	0.306
-No	36 (75)	41 (85.42)	
Burning sensation during sexual intercourse			
-Yes	1 (2.08)	4 (8.33)	0.168
-No	47 (97.92)	44 (91.67)	

^*^Statistically significant difference

### Histological assessment

**Fig. 6:**
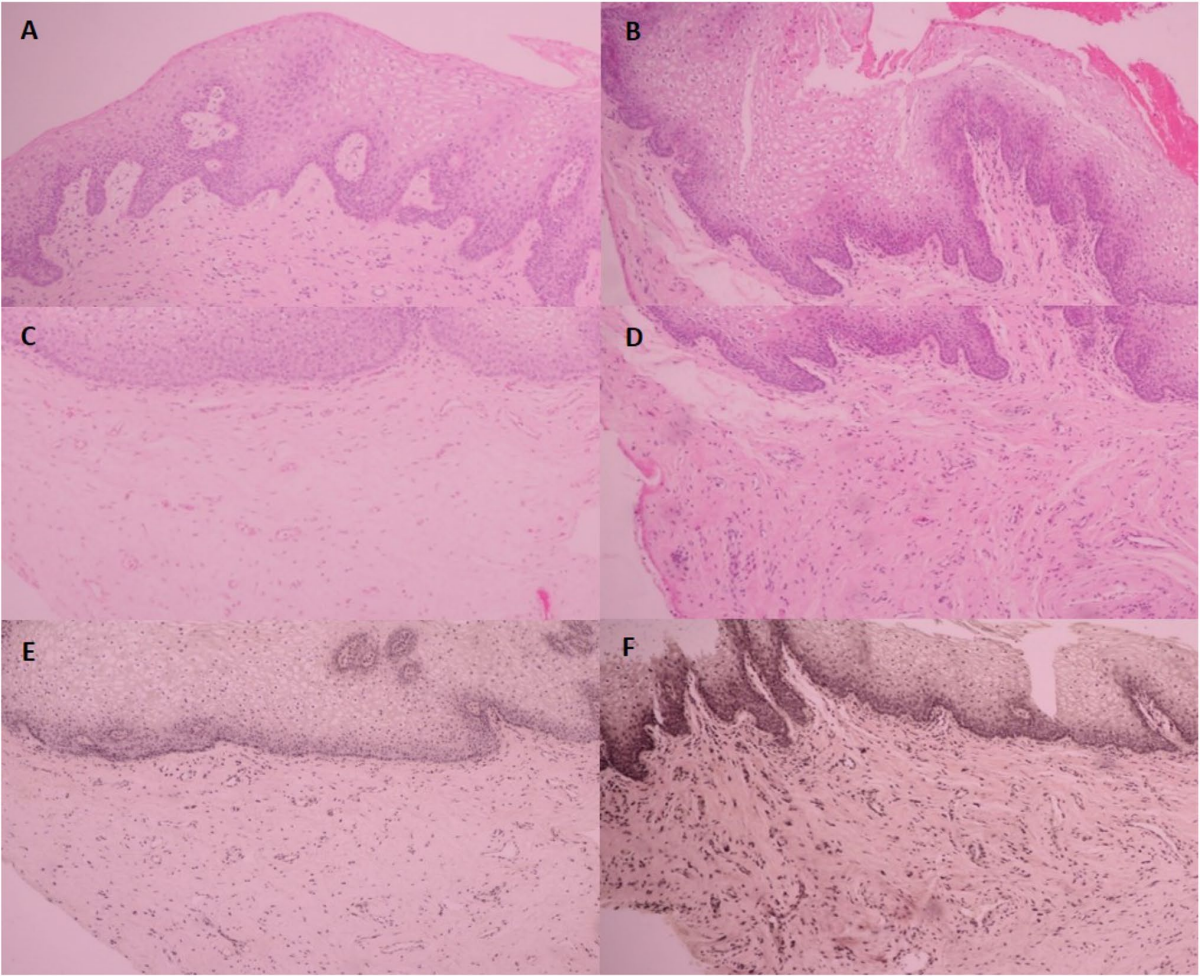
Histopathology of active RF + PEMF group. **A-D** Hematoxylin and eosin-stained sections. **A** Baseline vaginal epithelium. **B** 12 weeks after treatments showed thicker vaginal epithelium. **C** Baseline lamina propria. **D** Twelve weeks after treatments showed more compact lamina propria and denser arrangement of connective tissue with an increased number of blood vessels. **E–F** Verhoeff-van Gieson elastic staining. **E** Baseline elastic tissue. **F** Twelve weeks after treatments showed increased number in elastic tissue

**Fig.7:**
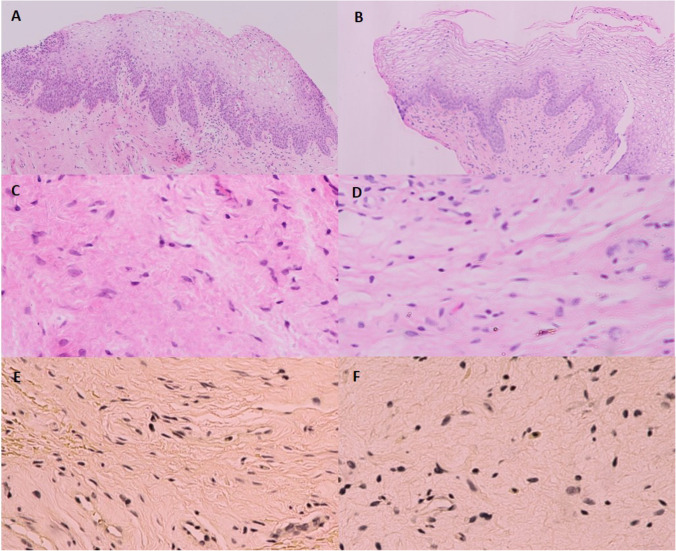
Histopathology of Sham group. **A-D** Hematoxylin and eosin-stained sections. **A** Baseline vaginal epithelium. **B** Twelve weeks after treatments showed no change of vaginal epithelial thickness. **C** Baseline lamina propria. **D** Twelve weeks after treatments showed loose arrangement of connective tissue with no change in number of blood vessels. **E–F** Verhoeff-van Gieson elastic staining. **E** Baseline elastic tissue. **F** Twelve weeks after treatments showed less elastic tissue

Vaginal biopsies pre and post-treatments were evaluated in 6 subjects (3 from each group). Hematoxylin–eosin stained sections in all 3 subjects receiving RF + PEMF treatment showed increased mucosal epithelial thickness, more compact lamina propria, and denser arrangement of connective tissue with an increased number of blood vessels (Figs. 6A-D). Changes in elastin content were evaluated using Verhoeff-van Gieson stain (shown in blue-black to black; Fig. 6E and 6F). The elastic tissue was increased and more prominent after the active treatments. Conversely, the histological findings in the sham group showed either decreased or similar degree of epidermal thickness, rete ridges, number of blood vessels, connective tissue, and elastin staining post-treatment (Figs. 7A-F).

## Discussion

Therapeutic options for vaginal laxity range from conservative pelvic floor muscle (Kegel) exercises and topical and systemic hormone replacement to non-invasive energy-based devices and surgical procedures [9–11]. Three non-surgical energy-based therapies have been developed for vulvovaginal rejuvenation: fractional CO2 laser, erbium:YAG laser, and RF. Each modality utilizes different mechanisms to penetrate and stimulate the vulvovaginal tissue. Non-ablative RF and microablative fractional laser treatments have recently gained popularity because of the minimal downtime, decreased risks, and side effects compared with invasive surgical modalities; however, well-designed, randomized controlled studies to confirm the safety and efficacy of these devices are lacking [13–16]. Currently, no device has been Food and Drug Administration (FDA) or European Medicines Agency (EMA) approved for the indication of treatment of VL or other genitourinary indications [29].

Radiofrequency are electromagnetic waves that generate heat upon meeting tissue impedance causing thermal injury of the tissue which activates micro-remodeling through heat shock protein cascades to stimulate connective tissue restoration with subsequent tissue tightening. RF devices have therapeutic applications in various aesthetic indications including facial rejuvenation, skin tightening, body contouring, cellulite, and non-invasive fat removal [24, 31–33]. Recently, RF has been used for vaginal rejuvenation; vaginal tightening; and in many other female genitourinary complaints such as stress urinary incontinence, genitourinary syndrome of menopause, and female sexual dysfunction [12–16, 23, 24, 30]. The principle of non-ablative RF treatment to improve VL is to induce heat in the vaginal wall to temperatures of 40 to 45 °C leading to fibroblast stimulation, collagen contraction, neocollagenesis, elastogenesis, vascularization, and growth factor production which play a role in restoring vaginal elasticity and increasing the moisture of vaginal mucosa. [13–16, 24]

This study is the first randomized, sham-controlled study to evaluate the efficacy and safety of a combination multipolar RF + PEMF therapy for the treatment of VL. Following 3 treatments results showed statistically significant improvements of self-reported vaginal laxity (VLQ), contraction pressure of the pelvic floor muscle, and overall sexual function (FSFI) with mild adverse effects. To the best of our knowledge, this is the second randomized, sham-controlled trial evaluating the effectiveness of RF treatment of VL in premenopausal women.

Significant improvements in the mean VLQ scores were achieved after RF + PEMF therapy compared to baseline (percentage improvement of 128% and 134% at 4- and 12-weeks post-treatments respectively) which were significantly greater than sham scores at both visits. Fifty percent of subjects in the active group reported no laxity (VLQ score > 4) at the second follow-up compared to 12% in the sham group, although this did not reach statistical significance between groups. Nonetheless, all subjects that still reported laxity post-active treatments showed at least some degree of VLQ improvement over baseline, whereas 8 subjects (50%) in the sham group showed no improvement. These results are similar to the findings in a previous sham-controlled study evaluating a surface-cooled monopolar RF device for VL in premenopausal women. Six months after one RF session, 43.5% of subjects reported no laxity compared to 19.6% receiving sham [24].

The study outcomes obtained by objective perineometer and Brink assessments of voluntary pelvic muscle contractions verified the patients’ self VLQ assessments. Maximum and mean contraction pressure of pelvic floor muscle contractions at both follow-up visits were significantly better in the active group compared to sham, and improvements over baseline were also significantly increased only in the active arm. Digital palpation Brink score assessment has been shown to correlate with perineometer measurements of pelvic floor muscle strength [27, 34]. Perineometer measurements in this study also confirmed the Brink assessments performed by a blinded nurse in which the active group achieved a 25% reduction in the number of weak score range and almost 20% increase in the very strong score range over baseline at 12 weeks post-treatment, while the sham group demonstrated no change in the percentage of subjects with weak scores after treatment and only slight 6.25% improvement in the strong scores.

Although this study was not designed to assess the effects of the device to treat female sexual dysfunction, it is probable that the improvement of VL and new nerve formation resulting from combination of RF heat and PEMF could positively impact subjects’ sexual function. Score results for the combined FSFI domains showed significantly greater FSFI improvements after RF + PEMF compared to the sham group. However, there was also a smaller, but statistically significant, FSFI improvement from baseline that occurred in the sham group, which could be attributed to a possible placebo effect. Placebos have been shown to improve outcomes, and placebo effects have been established in studies investigating treatment for female sexual dysfunction [35–38]. Previously, the sham effect has been noted in RF treatment on sexual function in women with VL, arising as a short-lasting effect which was unexpectedly high at 30% and 33% at 1 and 3 months post-intervention, respectively [24]. This emphasizes the strengths of this study which included a comparative sham group to substantiate that treatment outcomes are attributed to the active RF + PEMF intervention rather than to biases related to patients’ or investigators’ expectations of therapy. Despite the significant improvement of FSFI scores over baseline in this study, further studies enrolling women with VL and a baseline FSFI total score ≤ 26.5 indicating sexual dysfunction are potentially needed to establish the impact of RF + PEMF on sexual function.

The positive results from this study established in the active group over sham therapy support the proposed mechanism of action and safety profile of combined RF with PEMF delivery to the vaginal tissue. The RF induces the generation of bulk heat to 40–45 °C without tissue damage enhancing the structural integrity of mucosal squamous epithelial, while tightening the connective tissue in lamina propria and stimulating fibroblasts to produce new collagens and elastic fibers as was histologically confirmed in this study. To our knowledge, this study is the second to confirm the histologic changes in human vaginal tissue after RF treatment. The previous histology study performed in five females with VL who underwent pre- and post-treatment biopsies of the labia majora and vaginal canal 60 days after 3 transcutaneous temperature-controlled RF (TTCRF) treatments at 4-week intervals demonstrated post-TTCRF neocollagenesis, neoelastogenesis, and neoangiogenesis and was the first to report TTCRF-related neurogenesis [39]. Additionally, the nonrandomized vaginal TTCRF treatments resulted in significant improvements in vulvovaginal laxity, atrophic vaginitis, and sexual satisfaction. The authors proposed that improvements in orgasmic function and sexual satisfaction could be explained by the theory of thermal neurogenesis, in which repeated heat stimulation may induce neurite outgrowth [40].

PEMF also helps in increasing collagen synthesis by altering nitric oxide (NO) metabolism, which can induce an anti-inflammatory response via increased lymph and blood flow. NO also regulates cyclic guanosine monophosphate (cGMP) production which stimulate the release of relevant growth factors including fibroblast growth factor-2 (FGF-2), tumor necrosis factor alpha (TNFα) for collagen synthesis, vascular endothelial growth factor (VEGF) for angiogenesis, and transforming growth factor beta (TGF-β) for tissue remodeling [18, 21, 22, 31]. These underlying architectural modifications may improve the underlying structures including the vaginal canal and the circumferential of clitoral complex, hence enhancing genital arousal and potentially improved sexual function as observed in the FSFI score improvement in this study. In addition, the improvement in our patients’ sexual satisfaction might be explained by the aforementioned temperature-controlled repeated thermal stimulation from RF which has been shown to promote restoration of the nervous system and induce the new nerve formation [39, 40]. Additionally, a study of the regulation of neuritogenesis by a pulsed electromagnetic field suggests that PEMF stimulation independently induced neuronal repair [41]. Thus, simultaneous treatment with combination RF + PEMF in this device may have synergistic effects not only on tissue remodeling, neocollagenesis, and angiogenesis but also on neurite formation which may further benefit in female sexual function. Interestingly, a clinical study on the effects of repetitive, cumulative exposure to low-frequency PEMF in treatment of neuropathic pain was shown to quantitatively increase nerve fiber density and reduce itching but not pain which correlates to the adverse effects reported in this study in which the active group receiving PEMF had significantly less vaginal itching than the sham [42]. PEMF therapy has been used to manage postsurgical pain and edema, chronic wounds, and neuropathic pain. Therefore, it is possible that combination RF + PEMF may cause less pain and side effects than other RF devices. In this study, RF + PEMF treatment associated pain was minimal (VAS pain scores 0.50 out of 10). In comparison with a study utilizing a low energy monopolar RF device for VL treatment, the mean pain score was 1.5, and side effects included post-procedure vaginal leukorrhea and lower abdominal pain none of which were reported in this study [23]. Adverse effects in another study using monopolar RF therapy with cryogen surface cooling for treatment of VL were reported in 32.5% of 117 subjects, and the most common was vaginal discharge. Additionally, one treated subject experienced treatment pain that warranted discontinuation of the procedure [24].

The difference of this multipolar device over the monopolar devices is in how the electric current passes from the device through the tissue between the electrodes, with monopolar systems penetrating most deeply causing the possibility of hot-spot formation and more pain or adverse effects [31, 32]. This RF + PEMF device also allows for greater ease of use for the treatment provider since the internal vaginal applicator is maintained in stationary positioning during the 15-min treatment time. This is in contrast with the monopolar handpiece designs such as the cryogen-cooled RF [12, 23, 24] and the transcutaneous temperature-controlled RF device [43] that require introital rotations or sweeping movements passed over the multiple treatment zones while avoiding the urethral area with longer treatment durations of approximately 30 min.

The strengths of this randomized, clinical study included the double-blinded design in which both subjects and investigators were blinded of the interventions performed by therapists not involved in any other parts of the study and the inclusion of the sham arm to eliminate the potential placebo response after procedures [37, 38]. However, the small number of subjects and relatively short follow-up period (12 weeks) were the key limitations to this study. Given the ongoing effects of natural aging and other potential contributing factors (further childbearing, etc.), subsequent tissue changes could occur outside the time period analyzed in the study. It is warranted to have a longer follow-up period to fully assess the longevity of these results.

## Conclusions

This is the first randomized, sham-controlled, double-blinded clinical study using combined multipolar radiofrequency and pulsed electromagnetic energies for vaginal tightening in females experiencing vaginal laxity after vaginal deliveries. Three treatments with this device demonstrated significant objective and subjective improvements of VL with positive histological changes. Treatments were well tolerated with a favorable safety profile during the 18-week study period.
